# Targeting ferroptosis for treating kidney disease

**DOI:** 10.1007/s10157-024-02491-w

**Published:** 2024-04-22

**Authors:** Eikan Mishima

**Affiliations:** 1 Division of Nephrology, Rheumatology and Endocrinology, Tohoku University Graduate School of Medicine, Sendai, Japan; 2 Institute of Metabolism and Cell Death, Helmholtz Zentrum München, Neuherberg, Germany

**Keywords:** Cell death, Lipid peroxidation, Oxidative stress, Lipid radicals, Warfarin, Ischemia reperfusion injury

## Abstract

Ferroptosis is a type of regulated cell death hallmarked by iron-mediated excessive lipid oxidation. Over the past decade since the coining of the term ferroptosis, advances in research have led to the identification of intracellular processes that regulate ferroptosis such as GSH-GPX4 pathway and FSP1-coenzyme Q_10_/vitamin K pathway. From a disease perspective, the involvement of ferroptosis in pathological conditions including kidney disease has attracted attention. In terms of renal pathophysiology, ferroptosis has been widely investigated for its involvement in ischemia–reperfusion injury, nephrotoxin-induced kidney damage and other renal diseases. Therefore, therapeutic interventions targeting ferroptosis are expected to become a new therapeutic approach for these diseases. However, when considering cell death as a therapeutic target, careful consideration must be given to (i) in which type of cells, (ii) which type of cell death mode, and (iii) in which stage or temporal window of the disease. In the next decade, elucidation of the true involvement of ferroptosis in kidney disease setting in human, and development of clinically applicable and effective therapeutic drugs that target ferroptosis are warranted.

## Introduction

### Cell death in kidney disease

**Fig. 1:**
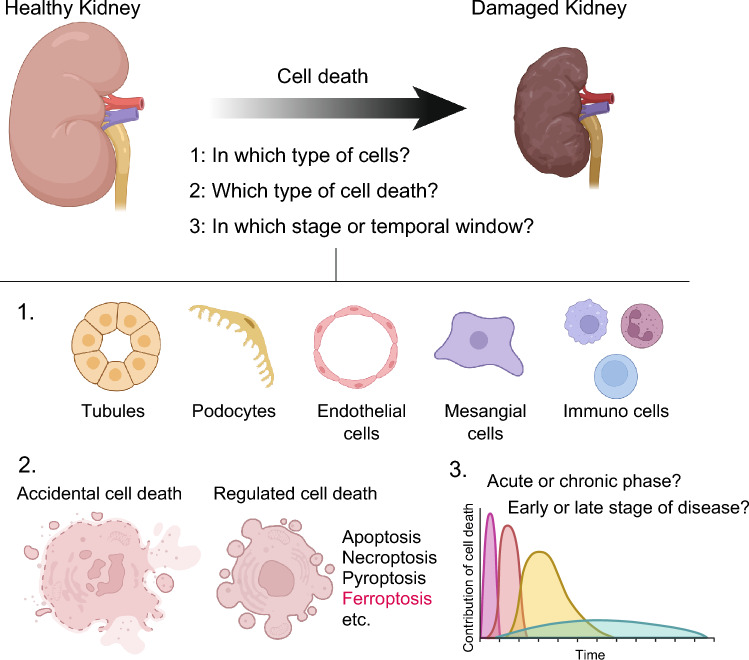
Targeting cell death for treating kidney diseases. Developing therapeutic interventions for kidney disease necessitates a comprehensive understanding of the specific cell death processes occurring in each component cell type and their implications for disease etiology. When considering cell death as a therapeutic target, careful consideration must be given to (1) in which type of cells, (2) which type of cell death mode, and (3) in which stage or temporal window cell death is involved in the disease pathophysiology

In kidney disease, whether acute or chronic, histologic changes occur in the affected cellular components of the kidney, including tubular epithelial cells, podocytes, endothelial cells, mesangial cells, etc. Acute kidney injury (AKI) often involves the disruption of renal tubular epithelial cells. In long-term disease conditions such as chronic kidney disease (CKD), kidney atrophy and nephron loss typically occur, resulting in diminished kidney function. These histological alternations are the result of cell death or degeneration and compensatory reactions within the diverse cell types comprising the kidney. Therefore, developing therapeutic interventions for kidney disease necessitates a comprehensive understanding of the specific cell death processes occurring in each component cell type and their implications for disease etiology (Fig. 1).

Cell death was classically categorized into necrosis and apoptosis. However, this classification is a past concept. In the current understanding, cell death is categorized into accidental cell death and regulated cell death [1] (Fig. 1). Accidental cell death results from uncontrolled events, such as mechanical disruption of cell membranes due to excessive physical damage, chemical exposure or heat. In contrast, regulated cell death is orchestrated through intracellular signaling involving proteins and genes dedicated to cell death execution, with a programmatically controlled process. Apoptosis is a representative example of this regulated cell death, but it includes many other modes of cell death. Necrosis, which involves rupture of the plasma membrane, was thought to be unregulated, but advance in cell death research have revealed regulated forms of necrosis, including necroptosis, pyroptosis, and ferroptosis. These forms of regulated necrosis are now considered part of the regulated cell death framework. Each type of regulated cell deaths plays distinct physiological role or is implicated in diverse pathological conditions.

### Ferroptosis

**Fig. 2:**
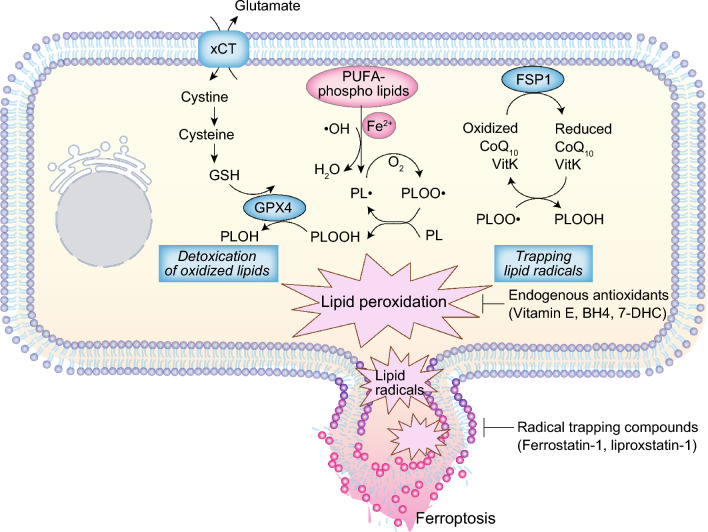
Ferroptosis, hallmarked by extensive lipid peroxidation, is considered to be initiated by Fenton reaction involving hydroxyl radical (•OH), which generates phospholipid radical (PL•) from a polyunsaturated fatty acid (PUFA)-containing phospholipids (PL), the primary constituents of lipid bilayers in cell membranes. Interaction with molecular oxygen (O_2_), PL• generates a phospholipid peroxyl radical (PLOO•). PLOO• react with another PUFA-PL, leading to the formation of phospholipid hydroperoxide (PLOOH), which is potentially toxic oxidized lipids. Extensive peroxidation of PL and the generation of lipid radicals, such as PLOO•, disrupt plasma membrane integrity, eventually triggering ferroptosis. **Left** Cystine/GSH/GPX4 pathway. Glutathione peroxidase 4 (GPX4) utilizes glutathione (GSH) to efficiently detoxify PLOOHs, converting them into corresponding alcohol (PLOH). Through detoxification of potentially toxic PLOOHs, GPX4 prevents ferroptosis. GSH is synthesized from cysteine, which is sourced from cystine transported via the cystine/glutamate antiporter xCT. **Right** FSP1-CoQ_10_/vitamin K pathway. Ferroptosis suppressor protein 1 (FSP1) reduces coenzyme Q_10_ (CoQ_10_) and vitamin K (VitK) to their reduced forms. The reduced forms of CoQ_10_ and vitamin K trap lipid radicals, suppressing lipid peroxidation and ferroptosis. Endogenous radical trapping antioxidants, including vitamin E, tetrahydrobiopterin (BH4) and 7-dehydrocholesterol (7-DHC); and radical trapping compounds, such as ferrostatin-1 and liproxstatin-1, also prevent ferroptosis by trapping lipid radicals

**Fig. 3:**
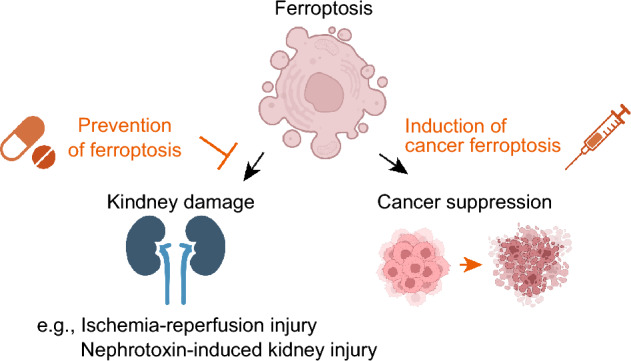
The role of ferroptosis in disease and the therapeutic strategy targeting ferroptosis. Ferroptosis plays a role in the pathophysiology of kidney damage (e.g., ischemia reperfusion injury and nephrotoxin-induced kidney injury), and cellular mechanisms involved in suppressing cancer progression. Therapeutic strategies involve the prevention of ferroptosis in kidney damage. In contrast, in the context of cancer, induction of ferroptosis is considered as a therapeutic strategy

Ferroptosis is a form of regulated cell death, characterized by excessive intracellular lipid oxidation [2]. In 2012, Dixon and Stockwell introduced ferroptosis as a new concept in cell death, emphasizing the involvement of iron in its triggering mechanism, as denoted by the prefix “fer-" indicating iron [3]. Subsequent investigations have elucidated that iron acts as a necessary molecule in facilitating lipid oxidation and/or lipid radical generation, and that excessive lipid oxidation is the hallmark feature of ferroptosis [4]. Unlike other cell death modalities, ferroptosis follows a distinctive execution pathway, wherein intracellular ion-mediated lipid oxidation progresses due to the disruption of the antioxidant defense system network that is supposed to inhibit lipid peroxidation. Consequently, excessive lipid oxidation generates lipid radicals that disrupt the cell membrane, resulting in ferroptotic cell death [5] (Fig. 2). A decade since its proposal, the major intracellular processes regulating ferroptosis have been unveiled [6]. In addition, ferroptosis has been implicated in diverse pathological conditions, including kidney diseases [7]. Consequently, therapeutic strategies targeting ferroptosis are gaining attention for these ferroptosis-associated diseases. In organ damage conditions, inhibiting ferroptosis holds promise, while in cancer, manipulating susceptibility to ferroptosis or promoting it in cancer cells are expected to be therapeutic avenues (Fig. 3).

### Ferroptosis regulation

A hallmark of ferroptosis is cellular excessive lipid oxidation triggered by the disruption of the antioxidant system crucial for preventing iron-mediated lipid oxidation. Thus, susceptibility to ferroptosis is mainly hinges on three key factors: (i) the efficiency of the antioxidant defense system against lipid oxidation; (ii) the composition of lipids in the plasma membrane; and (iii) the intracellular pool of free iron.

The cystine/glutathione/glutathione peroxidase (GPX4) pathway emerges as the prime defense mechanism against ferroptosis (Fig. 2). GPX4, which is the master regulator of ferroptosis, detoxifies oxidized lipids to corresponding alcohol by utilizing reduced glutathione (GSH) as cofactor [8, 9]. Since sufficient amounts of GSH are required for the optimal GPX4 activity, the GSH synthesis pathway is also important in the control of ferroptosis. Cysteine, a crucial amino acid for GSH biosynthesis, is taken up into cells as the oxidized dimer form cystine through the cystine/glutamate antiporter (xCT) [10]. Cystine is then intracellularly converted into the reduced from cysteine, which is used for GSH biosynthesis. Thus, cystine/GSH/GPX4 axis acts as a primary pathway for ferroptosis prevention. Recent findings have identified additional ferroptosis protective pathways, operating independently of GPX4. Ferroptosis suppressor protein 1 (FSP1), encoded by *AIFM2* gene, function as a reductase for coenzyme Q_10_ (CoQ_10_) located outside the mitochondria. The reduced form of CoQ_10_, mediated by FSP1, exerts its anti-oxidative effect as a scavenger of lipid radicals, preventing ferroptosis (Fig. 2) [11, 12]. The following study has reported that FSP1 also reduced vitamin K and FSP1-mediated vitamin K reduction acts as a potent ferroptosis suppressor [13]. In addition, endogenous antioxidants with lipid radical scavenging capacities, such as vitamin E, tetrahydrobiopterin, and 7-dehydrocholesterol, also contribute to ferroptosis suppression by inhibiting lipid oxidation [14–18].

The lipid composition of the plasma membrane also plays a crucial role in ferroptosis susceptibility since phospholipids containing polyunsaturated fatty acids (PUFAs) is more prone to lipid oxidation compared to saturated and monounsaturated fatty acids [19]. Thus, the PUFA content in the phospholipid bilayer becomes a determinant of ferroptosis susceptibility [20, 21]. Iron, an essential trace element, is indispensable for ferroptosis execution. Oxidized lipids react with free iron ions, generating lipid radicals and promoting lipid peroxidation chain reactions (Fig. 2). Consequently, various factors regulating cellular free iron levels, including iron uptake, excretion, storage and degradation, influence cellular susceptibility to ferroptosis [22].

### Ferroptosis and kidney disease

Mounting evidence underscore the role of ferroptosis in various kidney disease, including renal ischemia reperfusion injury (IRI), nephrotoxin-associated AKI, and CKD, suggesting that ferroptosis may emerge as a novel therapeutic target for these kidney diseases [7].

#### Ischemia reperfusion injury and ferroptosis

IRI is an event that causes acute organ damage upon the resumption of blood flow following an ischemic episode in an organ, leading to the generation of large amounts of free radicals and oxidative stress [23, 24]. In the context of the kidney, IRI commonly occurs after kidney transplantation or cardiac surgery, representing a major cause of AKI [25]. In addition, IRI serves as a widely utilized experimental animal AKI model [26]. Although acute tubular necrosis is the main pathological finding of renal IRI [27], surprisingly, no definitive conclusion has been reached as to the mode of cell death in its pathogenesis. This is because, for example, the involvement of apoptosis in necrotic tubules in IRI has recently been questioned. In the past, necrotic tubules were considered to be “apoptosis” because of positivity of terminal deoxynucleotidyl transferase dUTP nick end labeling (TUNEL) staining, which detects DNA double-strand breaks that occur during apoptosis [28] However, TUNEL staining, once considered an indicative of apoptosis, is now recognized to also mark other forms of regulated cell death, including ferroptosis [28]. Indeed, immunohistology with activated caspase-3 antibody, a specific marker of apoptosis, is minimally detected in necrotic tubules in IRI kidney [7], although activated caspase-3 positive cells can be found in inflammatory cells infiltrating at the injury site in IRI kidney. Notably, IRI occurs not only in the kidneys, but also in the liver, heart, brain, and other organs, causing acute organ damage with analogous findings. Similar to the kidney, IRI liver shows minimal percentage of active caspase-3 positive hepatocytes [13]. Therefore, it is now considered that apoptosis is actually less involved in cell death in the acute phase of IRI (at least in renal tubules and hepatocytes).

Conversely, a growing body of evidence supports the involvement of ferroptosis in renal IRI [29, 30]. For example, mice with an inducible whole-body knockout of the *Gpx4* gene exhibit AKI and succumb to ferroptosis in proximal tubule cells [8], underscoring the vulnerability of tubular cells to ferroptosis in vivo. Additionally, the administration of ferroptosis specific inhibitors, such as liproxstatin-1, ameliorated renal IRI [30] and mice lacking FSP1 and those with diminished GPX4 function display increased susceptibility to kidney damage in IRI [31]. Increased lipid peroxidation, a hallmark of ferroptosis, is observed in tubules within IRI kidneys [32, 33], further aligning with the pathological involvement of ferroptosis in renal IRI. Similar features have been noted in IRI affecting organs beyond the kidney, emphasizing the involvement of ferroptosis in IRI [8, 13]. However, since there is still no specific single molecular marker directly detecting ferroptosis, further studies are needed to definitively establish the contribution of ferroptosis to tubular cell death in renal IRI. In other experimental AKI conditions, ferroptosis inhibitors have demonstrated protective effects against tubular damages in rhabdomyolysis-associated AKI, folic acid-induced kidney injury, and oxalate-induced kidney injury [30, 34, 35]. In addition to tubular epithelial cells, ferroptosis inducers also induce ferroptosis in cultured cells derived from podocytes and renal fibroblasts [36]. However, the susceptibility of these kidney component cells to ferroptosis in in vivo setting, aside from tubular cells, remains elusive. A study utilizing tissue-specific conditional knockout of *Gpx4* gene in experimental animals would provide deeper insight into the unexplored topic.

#### Ferroptosis in CKD

The implication of ferroptosis in renal injury, extending beyond AKI, is gaining increasing attention. A study employing single-cell analysis of IRI kidneys has revealed that signals promoting ferroptosis, such as decreased expression of GPX4, play a role in fibrosis resulting from maladaptive repair of injured tubules, thereby contributing to the transition from AKI to CKD [32]. Another investigation, integrating genetic data from genome-wide association studies, identified dipeptidase 1 (DPEP1) and charged multivesicular body protein 1 A (CHMP1A) as genes associated with renal function. These genes were reported to contribute to the susceptibility to ferroptosis, suggest genetic variations, particularly single nucleotide polymorphisms, in these genes may contribute to the pathogenetic susceptibility of CKD [37]. Furthermore, recent studies using animal models have unveiled the involvement of ferroptosis in the pathogenesis of cystic kidney diseases and CKD-related arterial calcification [38, 39]. These investigations have not only highlighted the role of ferroptosis in these conditions, but also underscored the potential therapeutic benefit of inhibiting ferroptosis.

### What is the physiological role of ferroptosis?

While the pathological implications of ferroptosis in various disease conditions have been explored, the question regarding the physiological role of ferroptosis remains unanswered. From a developmental perspective, there is a study indicating a necessity for ferroptosis in embryonic erythropoiesis [40]. From an inflammatory perspective, in contrast to other regulated cell deaths, such as necroptosis and pyroptosis, which are highly immunogenic and induce inflammation [41] the influence of ferroptosis on immune system remains a subject of debate. A study has reported the less immunogenicity of ferroptotic dead cells despite the rupture of cell membranes during ferroptosis, leading to the release of inflammatory mediators [42]. Studies have suggested that a specific type of oxidized phospholipids and prostaglandins, which increase during the process of ferroptosis, exert anti-inflammatory activity [43, 44]. However, the precise contribution of ferroptosis in the inflammatory response are yet to be fully elucidated, emphasizing the need for research to unravel the complexity of ferroptosis in the context of inflammation.

### Pharmacological intervention for ferroptosis

As attention has focused on the link between ferroptosis and disease, there is a growing interest in developing compounds that serve as inhibitors or inducers of ferroptosis. To inhibit ferroptosis, several strategies have emerged, including (i) trapping lipid radicals using antioxidant compounds with lipid radical scavenging ability (e.g., ferrostatin-1 and liproxstatin-1) (Fig. 2); (ii) depletion of free iron through the use of iron chelators; and (iii) upregulation of ferroptosis defense pathways such as GPX4 and FSP1 [6]. Our research has demonstrated that several clinically available drugs and hormones with antiferroptotic properties, including rifampicin, promethazine, phenothiazine compounds, indole-3-carbinol, carvedilol, propranolol, estradiol, and thyroid hormones [36]. These drugs demonstrated antiferroptotic effects, exhibiting protective activities across various kidney cell types, including tubules, podocytes, and renal fibroblasts. Furthermore, in murine models, these drugs ameliorated AKI and liver injury associated with ferroptosis.

Conversely, to induce ferroptosis, pharmacological or genetic interventions disrupting the cystine/GSH/GPX4 pathway are mainly employed. Experimental agents such as RSL3 (a GPX4 inhibitor) [9, 45], erastin (an xCT inhibitor) [3] and BSO (which depletes GSH) are commonly utilized to induce ferroptosis. Since genetic knockouts of *Gpx4* also trigger ferroptosis, organ-specific knockouts of *Gpx4* are employed in animal models of ferroptosis [13, 46]. Inhibitors of FSP1 can enhance cell sensitivity, including in cancer cells, to ferroptosis, suggesting their potential development as anticancer drugs [11] (Fig. 3). We have developed novel FSP1 inhibitors, such as viFSP [47], the first species-independent FSP1 inhibitor, and icFSP1 [48], which alters intracellular FSP1 localization via phase separation, sensitize various cancer cell lines to ferroptosis. Additionally, our research revealed that brequinar, a dihydroorotate dehydrogenase (DHODH) inhibitor, functions as an FSP1 inhibitor at high concentration [49]. While DHODH inhibitors, including brequinar, were previously reported to sensitize to ferroptosis [50], our findings elucidate that their sensitizing effect on ferroptosis of these compounds is achieved not through DHODH inhibition but via off-target inhibition of FSP1.

### An unexpected link between vitamin K, warfarin, and FSP1

Vitamin K, a fat-soluble vitamin, plays a crucial role in blood clotting by acting as a cofactor for γ-glutamyl carboxylase (GGCX), which catalyzes the carboxylation of vitamin K-dependent proteins, including coagulation factors [51]. Our research has unveiled vitamin K as a potent inhibitor of ferroptosis [13]. During the screening for endogenous ferroptosis inhibitory metabolites, we unexpectedly discovered that vitamin K possesses ferroptosis inhibitory properties. Although the oxidized form of vitamin K, which is the normal existing form of vitamin K, has no antioxidant capacity, but the reduced form of vitamin K has potent antioxidant capacity and is capable of scavenging lipid radicals, thereby inhibiting ferroptosis. Notably, the enzyme responsible for the reduction of vitamin K, crucial for ferroptosis inhibition, was identified as FSP1, previously known as extramitochondrial CoQ_10_ reductase [11, 13]. In addition, pharmacological doses of vitamin K2 (menaquinone-4) were found to effectively ameliorate organ damage associated with ferroptosis induced by IRI and *Gpx4* knockout in the kidney and liver. Another research group similarly reported that administration of pharmacological doses of vitamin K1 alleviated renal IRI in mice [52].

**Fig. 4:**
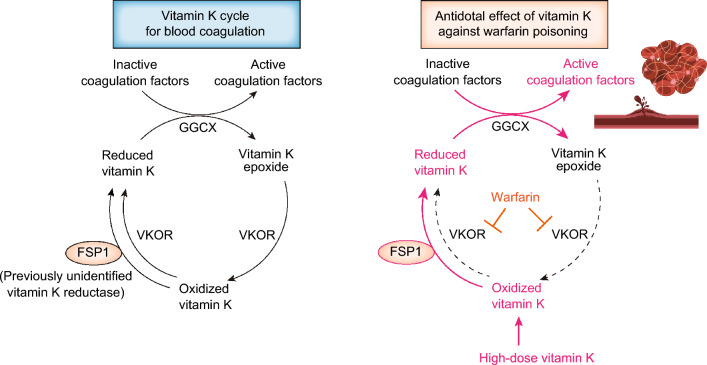
Vitamin K cycle for blood coagulation and antidotal effect of FSP1 against warfarin poisoning. **Left** Vitamin K cycle for blood coagulation. This cycle enables γ-carboxyglutamyl of vitamin K-dependent proteins, including coagulation factors (II, VII, IX, and X), and the recycling of vitamin K. The enzyme γ-carboxyglutamyl carboxylase (GGCX), in conjunction with reduced form of vitamin K as a cofactor, facilitates the activation of the coagulation factors. This reaction produces vitamin K epoxide, which is subsequently converted to oxidized form of vitamin K by vitamin K epoxide reductase (VKOR). Oxidized vitamin K is converted to the reduced from of vitamin K through either the warfarin-sensitive VKOR pathway, or the warfarin-resistant FSP1-mediated pathway. **Right** In the condition of warfarin overdose/poisoning, VKOR is completely inhibited, leading to the depletion of active coagulation factors and hypocoagulable state. Administration of a sufficient dose of vitamin K enables to provide the necessary amount of reduced form of vitamin K for the GGCX-mediated activation of the coagulation factors via the warfarin-resistant FSP1 pathway to bypass the inhibited VKOR pathway. This is the antidotal mechanism of vitamin K against warfarin poisoning

This discovery of the role of FSP1 in reducing vitamin K also unraveled a longstanding mystery—identifying the warfarin-resistant vitamin K reductase to combat anticoagulant warfarin poisoning (Fig. 4) [13]. Surprisingly, the molecular mechanism behind why vitamin K administration has antidotal effect on warfarin poisoning had remained elusive despite being a routine medical practice [53]. It was often misconceived that administering large doses of vitamin K compensates for the enzyme inhibition caused by an excess of the vitamin K antagonist warfarin. However, this notion is incorrect. Warfarin is a vitamin K epoxide reductase (VKOR), which stops vitamin K cycling. Thus, with excessive warfarin completely inhibiting VKOR, administered vitamin K cannot be converted to the reduced form of vitamin K, essential for activating coagulation factors. Therefore, another warfarin-insensitive vitamin K reductase was postulated to exist, but its molecule has not yet been identified over a half century [54, 55]. We identified FSP1 as this warfarin-insensitive vitamin K reductase, clarifying that the antidotal mechanism of vitamin K against warfarin overdose involves FSP1-mediated reduction of vitamin K, leading to the activation of coagulation factors even under warfarin poisoning [13, 51] (Fig. 4). Consequently, the identification of FSP1 as the warfarin-resistant vitamin K reductase elucidates the molecular mechanism of vitamin K’s antidotal effect against warfarin poisoning. In summary, FSP1 serves as a vitamin K reductase not only in the unconventional vitamin K cycle responsible for inhibiting ferroptosis (Fig. 2), but also in the conventional vitamin K cycle essential for the coagulation action of vitamin K (Fig. 4).

## Perspective and conclusion

Progress in the understanding of cell death mechanisms is unraveling the intricate impact of each form of cell death on the pathogenesis of kidney diseases. However, when considering cell death as a therapeutic target, careful consideration is required regarding (i) the specific mode of cell death; (ii) the stage or temporal window within the disease progression; and (iii) the precise cell type to target. In addition, since cell death processes may intricately regulate each other, the straightforward inhibition of a particular cell death modality may not present the optimal therapeutic solution against the complexity of diseases’ pathophysiology. Nevertheless, the control of ferroptosis is warranted as a promising target for kidney diseases.

When considering the development of clinically applicable drugs targeting ferroptosis, there are several issues to address. In experimental settings where ferroptosis is targeted for treatment, often radical trapping antioxidants (e.g., ferrostatin-1 and liproxstatin-1) are employed. However, reflecting on the history of drug development, many radical scavengers and antioxidants have proven ineffective or led to clinical application failures due to side effects (mainly liver damage resulting from radicalization of the compound themselves) although there are exceptions like edaravone, a radical scavenger approved for the treatment of amyotrophic lateral sclerosis [56]. Yet, with a clear understanding of ferroptosis as a specific target event, the development of safe radical trapping antioxidants with high in vivo efficiency targeting lipid radicals, based on compounds like liproxstatin-1 or vitamin K, is anticipated. In addition, rather than scavenging radicals with antioxidants, the development of drugs activating ferroptosis defense regulators, such as GPX4 and FSP1, can be considered as a strategy for pharmacological approach for ferroptosis prevention. Another issue for developing ferroptosis inhibiting drugs is the uncertainty regarding the long-term impact of ferroptosis prevention, especially when considering chronic diseases. Preventing ferroptosis in not only diseased cells, but also cancer cells might potentially have a risk of promoting cancer progression.

Over the past decade since the coining of the term ferroptosis, extensive research has elucidated the regulatory mechanisms and control strategies for ferroptosis. However, the reported involvement of ferroptosis in various pathological conditions, including kidney diseases, is primarily based on findings from animal studies. It is important to note that, as mentioned above, the absence of a definitive molecular marker poses challenges in reliably confirming the occurrence of ferroptosis in humans, particularly in disease states. The future trajectory of ferroptosis research is anticipated to uncover the specific disease conditions directly implicated in ferroptosis and identify disease states suitable for therapeutic interventions targeting ferroptosis. Moreover, translational research is expected to determine whether therapeutic intervention based on ferroptosis can truly be an effective therapy for disease. Therefore, in the next decade, we hope to elucidate the true involvement of ferroptosis in the human disease setting and to develop clinically applicable and effective therapeutic drugs that target ferroptosis.
